# The sero-prevalence of anti-adenovirus 5 neutralizing antibodies is independent of a chronic hepatitis B carrier state in China

**DOI:** 10.1007/s00705-015-2333-2

**Published:** 2015-01-24

**Authors:** Dao Huang, Marie Hennequi, Alexei Elvachev, Thierry Menguy, Nathalie Silvestre, DeMin Yu, Yue Han, Geneviève Inchauspé, Xinxin Zhang, Ren Zhu

**Affiliations:** 1 Department of Infectious Diseases, Institute of Infectious and Respiratory Diseases, Ruijin Hospital, Shanghai JiaoTong University School of Medicine, Shanghai, 200025 People’s Republic of China; 2 Transgene S.A. Boulevard Gonthier d’Andernach, Parc d’innovation CS80166, 67405 Illkirch-Graffenstaden Cedex, France; 3 Transgene S.A. Department of Infectious Diseases, Centre d’Infectiologie-Bâtiment Domilyon, 69007 Lyon, France; 4 Transgene Biopharmaceutical Technology (Shanghai) Co., Ltd, Shanghai, People’s Republic of China

**Keywords:** Adenovirus 5, Neutralizing antibodies, Chronic HBV infection

## Abstract

We investigated the prevalence of neutralizing antibodies (NA) to human Adenovirus (Ad) 5 both in healthy subjects (HS) and Chronic Hepatitis B (CHB) patients in Shanghai. Detection of anti-Ad5 NA (percentage of detection and titers) was similar between HS and CHB patients. A high percentage of subjects harbored no detectable antibodies (32.2 %) while proportion of subjects displaying very high antibody titers was low (4 %). Neither demographic factors (gender, age, health) nor AST/ALT or HBV circulating DNA titers affected detection of Ad5-specific NA. These observations pave the ground for development of Ad5-based immunotherapeutics aiming at treating CHB patients in China.

Adenovirus (Ad) vectors are extremely potent at inducing cellular-based immune responses to the encoded immunogens. They have been used to develop vaccines against a range of infectious diseases including human immunodeficiency virus (HIV), as well as cancers [1]. Ad serotype 5 has been the most widely used Ad vector because of earlier, historical development, its important safety profile and its remarkable capacity to induce strong, long-lasting and broad T-cell based immune responses, in particular CD8^+^ driven ones [2].

We have developed a novel immunotherapeutic TG1050 based on Ad5 that has been engineered to express three major antigens or antigenic domains encoded by the Hepatitis B virus (HBV) genome: the core, polymerase and HBsAg [3]. This novel therapeutics aims at improving treatment of Chronic Hepatitis B (CHB) patients in particular at increasing cure rate. TG1050 has been shown in pre-clinical studies to induce robust, broad, long-lasting as well as cross-reactive T cells displaying characteristics similar to those found in patients who resolve infection, together with early antiviral activity [3]. Because anti-vector pre-immunity may have some effects on immunity mounted against the vector-encoded immunogens, it is important to document level of vector pre-immunity in the vaccine-targeted population. A number of studies have documented important geographic variations in the sero-prevalence of Ad5 ranging from 40-70 % in the USA and Europe, to 90 % in various regions of Africa and 95% in Thailand [4]. Recent publications indicate a prevalence in the range of 60-70 % in the Chinese population [5–8]. Because Ad5-based immunotherapies may be of great value in the treatment of CHB in countries displaying elevated prevalence of Hepatitis B, it is important to evaluate whether infection by HBV may influence anti-Ad5 sero-prevalence.

Recently, Jian et al. assessed the prevalence of NA to chimpanzee adenovirus (AdC) serotypes 6 and 7 in healthy adults, CHB patients and patients with primary hepatocellular carcinoma (HCC) in China. They demonstrated that the sero-prevalence rate of AdC6 and AdC7 in CHB patients and HCC patients were much higher than in healthy subjects [9].

We compare here such prevalence of Ad5 NA in both healthy subjects (HS) and CHB patients in subjects living in Shanghai and vicinity.

Two hundred plasma samples from healthy donors, and 204 serum samples from age and gender matched CHB outpatients from Ruijin hospital (Shanghai, China) were collected. Subjects were divided into four age groups (See Table 1A and B). Clinical parameters including HBV viral load, Alanine transaminase (ALT) and Aspartate transaminase (AST) levels were collected.

**Table 1:** Sero-prevalence of Ad5 NA in healthy subjects (A) and CHB patients (B)

	**Ad5 NA titer, no. (%)**					
	**< 20**	**≥ 20**				**Overall**
		**20-200**	**201-1000**	**> 1000**	**Total (≥** **20)**	
**A.**						
** Overall**	73 (36.50)	46 (23.00)	73 (36.50)	8 (4.00)	127 (63.50)	200
**Age, years**						
**21-30**	20 (40.00)	14 (28.00)	13 (26.00)	3 (6.00)	30 (60.00)	50
**31-40**	21 (42.00)	9 (18.00)	17 (34.00)	3 (6.00)	29 (58.00)	50
**41-50**	16 (32.00)	8 (16.00)	25 (50.00)	1 (2.00)	34 (68.00)	50
**51-55**	16 (32.00)	15 (30.00)	18 (36.00)	1 (2.00)	34 (68.00)	50
**Sex**						
**Male**	37 (37.00)	23 (23.00)	39 (39.00)	1 (1.00)	63 (63.00)	100
**Female**	36 (36.00)	23 (23.00)	34 (34.00)	7 (7.00)	64 (64.00)	100
**B.**						
**Overall**	57 (27.94)	53 (25.98)	86 (42.16)	8 (3.92)	147 (72.06)	204
**Age, years**						
**21-30**	14 (28.00)	9 (18.00)	25 (50.00)	2 (4.00)	36 (72.00)	50
**31-40**	13 (26.00)	10 (20.00)	26 (52.00)	1 (2.00)	37 (74.00)	50
**41-50**	13 (25.49)	20 (39.22)	15 (29.41)	3 (5.88)	38 (74.50)	51
**51-55**	17 (32.08)	14 (26.42)	20 (37.74)	2 (3.77)	36 (67.92)	53
**Sex**						
**Male**	32 (31.07)	24 (23.30)	43 (41.75)	4 (3.88)	71 (68.93)	103
**Female**	25 (24.75)	29 (28.71)	43 (42.57)	4 (3.96)	76 (75.25)	101

Ad5-specific NA titers were measured using an adapted luciferase-based virus neutralization assay described by Sprangers et al. [10]. Briefly, diluted samples were mixed with 1.33 × 10^7^ viral particles (VP) of replication defective (E1 and E3 deleted) luciferase-expressing Ad vector (ShenZhao Biotechnology, China), and incubated for 1 hour at room temperature. Five × 10^4^ A549 target cells were added and incubated at 37 °C, 5 % CO_2_. Following 24 h incubation, luciferase activity in the cells was quantified using Luciferase Assay System (Promega) with a Microplate Reader (BioTek). The 90 % neutralization sample titer was determined to be the sample dilution which resulted in 90 % neutralization of the Ad-luciferase vector replication as tested on A549 cells. Ranges of titers were defined as < 20, 20-200, 201-1000, or > 1000. They were selected to give a qualitative representation of subjects displaying negative, low, moderate or high pre-existing anti-Ad5 immunity, respectively.

Due to the restrictions of sample collection, plasma samples were collected from HS, while serum samples were obtained from CHB patients. To exclude a potential effect of the sample type on the sensitivity of detection of Ad5 NA, we compared NA levels in serum or plasma of six subjects. As anticipated, no difference was observed (data not shown) supporting comparative analysis using either type of samples.

Sero-prevalence was reported based on age, gender and CHB infection status. In order to analyze impact of these parameters on NA titers, all participants were split into either negative (< 20) versus positive (≥ 20) groups or into negative and low titers (≤ 200) versus medium and high titers (> 200) groups. The distribution of patients in these groups according to each demographic parameter was tested using the Chi-Square test. Multivariate and univariate logistic regressions were performed to calculate odds ratios (OR) and 95 % confidence interval (CI) in each demographic subgroup. Spearman’s correlation coefficients were calculated to analyze correlation between clinical parameters (ALT, ALT and HBV-DNA) and Ad5 NA titers. Analyses were performed with the software SAS 9.3. P-values <0.05 were considered significant.

The prevalence of Ad5 NA in plasma samples taken from 200 HS in Shanghai from 20-55 years of age was evaluated. As shown in Table 1A, 63.5% of HS (127/200) displayed detectable anti-Ad5 NA (titers ≥ 20). Overall, 23 % (46/200) and 36.5 % (73/200) of participants displayed low (20-200) or moderate (201-1000) NA titers, respectively, while only 4 % (8/200) of individuals displayed high titers (>1000).

Anti-Ad5 sero-prevalence was concomitantly evaluated in 204 age and gender matched CHB outpatients. As shown in Table 1B, 72.06 % of CHB patients (147/204) displayed anti-Ad5-specific NA, a rate similar to that seen in HS. Twenty-six percent (53/204) and 42 % (86/204) of patients displayed low (20-200) and moderate (201-1000) levels of NA titers, while only 3.9 % (8/204) of them displayed high NA titers (> 1000).

When subjects were stratified on the basis of detection or not of Ad5 NA, i.e. whether they displayed titers < 20 or ≥ 20, no difference was detected between HS and CHB patients or between different age/gender groups (Table 2A). Similarly, when stratification was made on a broader basis, i.e., between subjects with negative or low titers ≤ 200 versus medium and high titers > 200, no statistical differences could be observed within the groups compared (Table 2B).

**Table 2:** Distribution of Ad5-specific NA titers

**A.** Distribution of Ad5 NA titers (< 20 vs ≥ 20) according to clinical status (HS, CHB), age and gender				
	**Ad5 NA titer, no. (%)**			
	**< 20**	**≥ 20**	**Overall**	P-value*
**Overall**	130	274	404	
**Clinical status**				0.066
**Healthy subjects**	73 (56.15)	127 (46.35)	200 (49.50)	
**CHB subjects**	57 (43.85)	147 (53.65)	204 (50.50)	
**Age, years**				0.841
**20-30**	34 (26.15)	66 (24.09)	100 (24.75)	
**31-40**	34 (26.15)	66 (24.09)	100 (24.75)	
**41-50**	30 (23.08)	74 (27.01)	104 (25.74)	
**51-55**	32 (24.62)	68 (24.82)	100 (24.75)	
**Gender**				0.475
**Male**	69 (53.08)	135 (49.27)	204 (50.50)	
**Female**	61 (46.92)	139 (50.72)	200 (49.50)	

**B.** Distribution of Ad5 NA titers (≤ 200 vs > 200) according to clinical status (HS, CHB), age and gender				
	**Ad5 NA titer, no. (%)**			
	**≤ 200**	**> 200**	**Overall**	P-value*
**Overall**	229	175	404	
**Clinical status**				0.258
**Healthy subjects**	119 (51.97)	81 (46.29)	200 (49.5)	
**CHB subjects**	110 (48.03)	94 (53.71)	204 (50.50)	
**Age, years**				0.800
**20-30**	57 (24.89)	43 (24.57)	100 (24.75)	
**31-40**	53 (23.14)	47 (26.86)	100 (24.75)	
**41-50**	59 (25.76)	45 (25.71)	104 (25.74)	
**51-55**	60 (26.20)	40 (22.86)	100 (24.75)	
**Gender**				0.784
**Male**	117 (51.09)	87 (49.71)	204 (50.50)	
**Female**	112 (48.91)	88 (50.29)	200 (49.50)	

**C.** Odds ratio and 95% confidence intervals of Ad5 NA titers (≤ 200 vs > 200) according to clinical status (HS, CHB), age and gender status, based on univariate and multivariate logistic regression		
	**Univariate analysis**	
**Effect**	**OR [95%CI]**	**P-value**
**Age**		
**Age (31-40 vs 21-30)**	0.85 [0.487 – 1.486]	0.390
**Age (41-50 vs 21-30)**	0.99 [0.568 – 1.722]	0.994
**Age (51-55 vs 21-30)**	1.13 [0.645 – 1.987]	0.440
**Sex**		
**Sex (Female vs Male)**	0.95 [0.638 – 1.403]	0.784
**Subject**		
**Clinical status (CHB vs HS)**	0.80 [0.537 – 1.182]	0.258

	**Multivariate analysis**	
**Effect**	**OR [95%CI]**	**P-value**
**Sex (Female vs Male)**	0.95 [0.637 – 1.403]	0.780
**Age (31-40 vs 21-30)**	0.85 [0.489 – 1.486]	0.386
**Age (41-50 vs 21-30)**	0.99 [0.570 – 1.730]	0.980
**Age (51-55 vs 21-30)**	1.13 [0.644 – 1.988]	0.444
**Health (CHB vs HS)**	0.80 [0.536 – 1.181]	0.256

*P-value based on chi-square test

A global statistical analysis was also performed to evaluate the impact of different demographic parameters including age, gender and CHB infection status on the sero-prevalence of anti-Ad5 NA (Table 2). Multivariate and univariate analyses of factors associated with Ad5 NA titers are shown in Table 2C with associated odds ratios (95 % CI) and p-values. None of the studied factors had any significant effect on the levels of detected Ad5 NA titers, either in subjects with titers < 20 versus ≥ 20 or in subjects with titers ≤ 200 versus > 200 (Table 2).

We examined whether there was a correlation between clinical parameters such as HBV-DNA load, ALT and AST levels (data not shown) and Ad5 NA titers in CHB patients. Spearman’s correlation coefficients were used and showed that levels of Ad5-specific NA titers were not significantly correlated with these clinical parameters (Fig. 1).

**Fig. 1:**
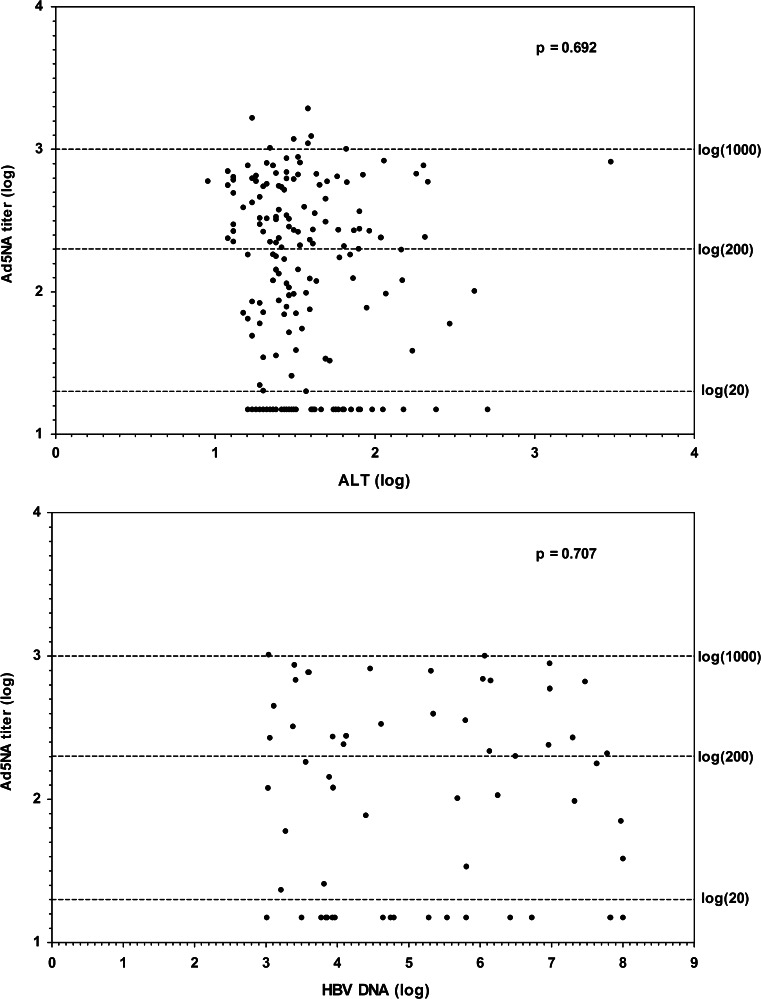
Impact of clinical parameters on Ad5 NA titers. NA titers to Ad5 were determined in sera from 204 CHB patients, and the correlation between Ad5 NA titers and ALT (Upper panel) and HBV DNA level (Bottom panel) was analyzed. Levels of Ad5 NA titers, ALT and HBV DNA were represented in logarithmic values (log). P values (based on Spearman’s correlation) are indicated on the graph

Adenoviruses and in particular human Ad 5, have been widely developed as vaccine vehicles because of their high safety profile and their unique capacity to induce strong T-cell based immunity [2]. In the field of HIV vaccine development, high levels of NA to Ad5 have been shown to lower detection of immunogen-specific induced immunity following vaccination and/or to restrict the scope of epitope detection [11, 12]. Contrary observations have been reported more recently in studies of Ad5-based anti-malaria and anti-tuberculosis vaccines [13, 14]. Although these later studies were of low size (phase 1), the authors reported a lack of effect of anti-Ad5 pre-immunity on induction of immunogen-specific T-cells, observations that are very encouraging for the development of non-HIV Ad5-based vaccines. In China, at least one Ad5-based immunotherapeutics has successfully reached the market, supporting the value of the Ad5 platform [15]. An international epidemiological study by Mast et al. showed that Ad5 NA titers vary according to geographic location, with titers being higher in non-US and non-European countries [4]. Recently, detailed studies have been reported on the sero-prevalence of Ad5 NA in China [5–8]. In particular, two studies have shown that 77.4 % of healthy adults in Guangzhou and 72 % of healthy adults in six different regions of China displayed detectable Ad5 NA [ 5,6]. In agreement with the results of these studies and others performed in China [5–7], we observed 63.5 % of sero-prevalence to Ad5 NA in 200 HS from Shanghai. In addition, we demonstrated that more than half (60%) of the tested HS have negative or low titers of Ad5 NA (≤ 200), while the proportion of high titers individuals (> 1000) was very low, only 4%. We confirmed that gender or age difference did not significantly impact the level and titers of Ad5 NA in HS. Because CHB infection is an important and well-recognized medical concern in China [16], efforts are ongoing to develop novel curative treatment regimens. We have developed a novel Ad5-based immunotherapeutic TG1050, which gathers important immunological features found in resolvers [3]. The present study shows that 72.06% of CHB patients displayed detectable anti-Ad5 NA titers, which, in their levels and distribution, are comparable to those seen in HS (*p* > 0.05).

We confirmed that a very small number of CHB patients (3.92 %) display high NA titers (> 1000). Interestingly, our study did not reveal any correlation with HBV-DNA, ALT, and AST levels and Ad5 NA titers. Overall, the collected data indicate that CHB infection does not influence sero-prevalence and titers of Ad5 NA, implying that chronic infection by HBV does not modulate neither positively nor negatively the development of NA to Ad5. Obviously, it would be of interest to extend our study to other regions in China, beyond the region of Shanghai.

It is difficult to strictly compare studies that have evaluated the presence and titers of Ad5 NA, as assays are not universally standardized and vary from study to study [5–8]. In our study, the proportion of individuals, either HS or CHB patients in Shanghai, displaying high titers of Ad5 NA, was lower than that reported by Yu *et al*. A number of reasons may explain the differences observed. Our studied population was likely different from that studied by Yu et al. [6], (different geographic location) and, for example, may have included more migrant population (Shanghai is known to have a large migrant population). It would be of obvious interest to expand our analysis beyond the area of Shanghai in order to generalize our observations to major cities in China. In addition, in our study, Ad5-specific NA titers were measured using chemiluminescence-based NA test (CLNT), which has higher sensitivity than the fluorescence-based NA (FRNT) assay reported by Liu et al. [8]. Our evaluation was primarily based on 90 % neutralization and not 50 % as often reported. Even when similar CLNT tests are used [7, 9], different protocols (such as virus concentration, cell density, etc.), use of different luminometers, and different definitions of neutralization titers may all influence the quality and sensitivity of detection assays, rendering comparisons delicate to perform.

In conclusion, our study shows that the sero-prevalence and titer of Ad5 NA are not affected by infection with HBV, an observation supporting the development of TG1050 in Chinese CHB infected patients.

## Abbreviations

Ad: Adenovirus
ALT: Alanine transaminase
AST: Aspartate transaminase
CHB: Chronic Hepatitis B
AdC: Chimpanzee adenovirus
HBV: Hepatitis B virus
HCC: Hepatocellular carcinoma
HIV: Human immunodeficiency virus
HS: Healthy subject
NA: Neutralizing antibodies
VP: Viral particle
